# Polymyositis and covid-19: A morbide association (a case report)

**DOI:** 10.1016/j.amsu.2021.102598

**Published:** 2021-07-26

**Authors:** L. Musoni, H. Ezzouine, O. Ettouki, A. Mansour, M. Nour, I. Elkhaouri, A. Darif, M. Raid, M. Elkasmi, B. charra

**Affiliations:** a Faculty of Medicine and Pharmacy, Hassan II University of Casablanca, Morocco; b Medical Intensive Care Unit, CHU Ibn Rochd, Casablanca, Morocco

**Keywords:** SARS-Cov-2, Polymyositis, COVID-19, Morbid association, Case report

## Abstract

The COVID-19 pandemic and its impact on health systems had a significant effect on the management of inflammatory diseases in the long term and myopathies could be signs of COVID-19, making it difficult to diagnose the cause and effect relationship.

An unvaccinated 62-year-old female patient followed for polymyositis was tested positive for COVID-19 on polymerase chain reaction (PCR) of nasopharyngeal swab revealed by dyspnea and rhinorrhea with fever and pulmonary involvement of 75%. She had an enlarged left ventricle with complete left branch block, inaugural diabetes mellitus with ketosis, kidney dysfunction, and inflammatory syndrome. Despite the early initiation of invasive ventilation in combination with the national protocol against covid-19, the patient died on day 4 of care. The best management should anticipate comorbidities and the evolutionary profile would guide the continuation of the treatment. Polymyositis like other rheumatic diseases was associated with a very high risk of developing a severe form of COVID-19. The combination of elder age and comorbidities led to a severe form of COVID-19 and therefore to a poor prognosis. The article aimed to show the severity of the association of covid-19 with polymyositis at the comorbid stage.

## Highlights

• The association of covid-19 and polymyositis is few or not frequent in the literature.
• Myositis can be one of the symptoms of covid-19 with risk of a worsening of the pre-existing pathology: polymyositis.
• The article shows the severity of the association of covid-19 with polymyositis.
• Poorly controlled treatment of polymyositis leads to serious consequences with the severe form of covid-19.
• Polymyositis is a risk factor for severe covid-19.

## Conflicts of interest

None.

## 1 Introduction

The COVID-19 pandemic and its impact on health systems had a significant effect on the management of inflammatory diseases in the long term. Inflammatory myopathies, of which polymyositis is a part, could be signs of COVID-19, making it difficult to diagnose the cause and effect relationship. The long-term treatment given to these patients exposes them to side effects that are sometimes fatal or may induce them to develop the severe form of COVID-19 disease. Patients with autoimmune and inflammatory diseases have a higher incidence of co-morbidities such as metabolic syndrome and ischemic heart disease putting them at risk of developing severe COVID-19 infection. The aim of this work was to show the severity of the association of covid-19 with polymyositis at the comorbid stage.

This manuscript has been reported in line with SCARE's 2020 Criteria [1].

## 2 Case report

We report a case of an unvaccinated 62-year-old female patient followed for polymyositis under corticosteroid therapy for seven years. Five days before admission the patient presented with dyspnea associated with rhinorrhea with uncalculated fever. When the respiratory symptoms worsened, the patient went to the emergency room with pulsed oxygen saturation at 40% in ambient air and 91% under a high-concentration mask. A chest CT scan with contrast injection showed multiple frosted glass foci of multilobar and bilateral pleural and central topography, some linear subpleural opacities without lymph node hypertrophy of the mediastinal and hilar chains, moderate cardiomegaly with the integrity of the pleura and the wall concluding to a COVID-19 viral pneumopathy with pulmonary involvement estimated at 75% (Fig. 1) which was confirmed with a polymerase chain reaction of the nasopharyngeal swab. On admission in the intensive care unit, she was conscious with a Glasgow coma scale of 15/15, reactive and symmetrical pupils, without sensory-motor deficit or cutaneous signs of dermatomyositis. Blood pressure at 140/70 mmHg, heart rate at 80 beats per minute, pulse oxygen saturation at 77% in ambient air and 93% under high concentration mask at 15L/min, and breathing rate at 22 cycles per minute (CPM). The electrocardiogram showed a regular sinus rhythm at 100 beats per minute, the PR space fixed at 0.16 sec; complete left branch block, QT corrected to 0.42sec. On transthoracic echocardiography, we noted an undilated left ventricle with minimal left ventricular hypertrophy, global contractility preserved with the ejection fraction estimated at 50%, minimal mitral insufficiency, complying with undilated inferior vena cava (17mm). Capillary blood glucose was at 27.5mmol/L, urinary labstix noted Ketone ++, glycosuria +++, glycated hemoglobin at 12.8%, which concluded to a diabetes mellitus with ketosis, probably induced by long-term corticosteroid therapy. Blood gases showed PH: 7.39; PaCO2: 44 mmHg; PaO2: 59.5 mmHg; HCO3-: 26.4mmol; TCO2: 27.8mmol; SaO 2: 90.5% under high concentration mask at 15L/minute; respiration rate 21 cpm with a ratio PaO2/FiO2 = 198.3. The biological tests show a blood count with white blood cells at 9840/mm3, neutrophil polynuclear cells at 7950/mm3, lymphocytes at 1130/mm3, blood platelets at 321000/mm3, hemoglobin at 12.2g/dl. In addition, *C*-reactive protein at 316 mg/L; blood urea at 14.65mmol/L; blood creatinine at 207.74μmolg/L; aspartate aminotransferase (ASAT) at 44UI/L; alanine aminotransferase (ALAT) at 22UI/L; Albumin at 36g/L. The patient received a nationally adopted treatment for SARS-CoV-2 made by hydroxychloroquine, azithromycin, vitamin C & D; zinc, ceftriaxone, low molecular weight heparin preventive dose, acetylsalicylic acid; methylprednisolone, insulin therapy, proton pump inhibitor as well as respiratory physiotherapy. The evolution was marked by the worsening on the respiratory level with Pulse Oxygen Saturation at 88% under high concentration mask at 15L/min, a polypnea at 30 cpm, and 97% under non-invasive ventilation with polypnea at 24 cpm. The patient was intubated on day 3 and died on day 4 of hospitalization due to severe refractory hypoxia.

**Image:**
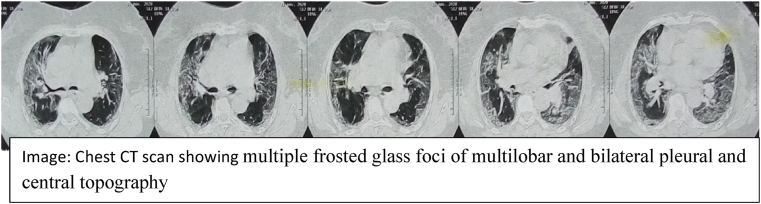
Chest CT scan showing multiple frosted glass foci of multilobar and bilateral pleural and central topography.

## 3 Discussion

Polymyositis is a rheumatic disease of inflammatory myopathies [2]. It is clinically characterized by proximal muscle weakness of the limbs with myalgia [3], and when it affects the respiratory system, it can manifest itself as cough, fever, and dyspnea. The biological inflammatory syndrome can vary, and the creatine kinase assay confirms the myogenic syndrome with a level greater than or equal to three times normal [4]. Treatment is mainly based on corticosteroids and immunosuppressants, and the choice of treatment is guided by the presentation of the disease [3,4]. In the absence of underlying tumor pathology, adult myositis has a relatively favorable prognostic condition, with current 5-year survival rates of about 90% [4]. Substantive treatment must be continued with an adaptation of corticoid doses, avoiding non-steroidal anti-inflammatory drugs, and favoring paracetamol for symptomatic treatment [5,6]. It should also be mentioned that patients with rheumatic diseases are more sensitive to COVID-19 either because of the rheumatological disease complication or because of the drugs used [6,7]. Patients with myositis, dermatomyositis, polymyositis have a very high risk of developing COVID-19 [5]. The immune response is compromised for patients under immunosuppressive therapies, and then are more exposed to infections [8]. Other Risk factors for developing severe COVID-19 infection in rheumatic patients are elder age, lung disease, diabetes mellitus, long-term corticosteroid therapy, sepsis, high activity of the underlying rheumatic disease [5],cardiac pathologies, pneumopathy [4,7], associated tumor pathology, black race, delay of inadequate initial therapy [4]. Our patient was being followed for polymyositis under long-term corticosteroid therapy. She presented a fever with dyspnea and rhinorrhea with a biological inflammatory syndrome made of a *C*-reactive protein at 316mg/L. The lung involvement was estimated at 75% on a chest CT scan. It concluded to SARS COV-2 on polymyositis with comorbidities. It has been described that polymyositis could be observed during infection with viruses, especially retroviruses [4] and more recently COVID-19 [9]. Comorbidities such as diabetes mellitus, heart disease, obesity, impaired renal function are risk factors for mortality [10,11], and when associated with an age greater than 59 years, are predictive factors for the development of rapidly progressive severe or fatal COVID-19 infection [[10], [11], [12], [13]].

## 4 Conclusion

Patients followed for polymyositis are exposed to a very high risk of developing a severe form of COVID-19. Therapeutic management of COVID-19 remains the same as for other patients. The treatment of the comorbidities is a cornerstone of a good prognosis. Despite the early diagnosis and treatment, the prognosis of these patients remains poor and putting polymyositis in the risk factors of severe forms of covid-19.

## Ethical approval

The study is exempt from ethical approval in our institution.

## Author contribution

Libérat Musoni: designed the study, wrote the protocol and the first draft of the manuscript Hanane Ezzouine: designed the study, wrote the protocol and the first draft of the manuscript Omar Ettouki: designed the study, wrote the protocol and the first draft of the manuscript Akram. Mansour: managed the analyses, and the correction of the manuscript Mariam Nour: managed the analyses, and the correction of the manuscript Imane Elkhaouri: managed the analyses, and the correction of the manuscript Aicha Darif: managed the analyses, and the correction of the manuscript Mehdi Raid: managed the analyses, and the correction of the manuscript Maroua Elkasmi: managed the analyses, and the correction of the manuscript Boubaker charra: reading and correction of the manuscriptAll authors read and approved the final manuscript.

## Sources of funding

No funding for research.

## Registration of research studies: not necessary it is not the first case

• Clinicaltrials.gov – for all human studies – free.

• Chinese Clinical Trial Registry chictr.org.cn – for all human studies - free.

• Researchregistry.com – for all human studies – charge.

• ISRCTN.com – for all human studies – charge.

• There are many national registries approved by the UN that can be found here.

## Guarantor

MUSONI Libérat.

## Consent

Written informed consent was obtained from the patient's family for publication of this case.

## Declaration of competing interest

No conflicts of interest.
